# UiO-66-NH_2_@纤维素复合气凝胶用于保健品中西地那非的固相萃取

**DOI:** 10.3724/SP.J.1123.2021.11022

**Published:** 2022-06-08

**Authors:** Zhifan CHEN, Yeyu WU, Xuecai TAN, Jianqing MENG, Jie CEN, Min LIU

**Affiliations:** 广西民族大学化学化工学院, 林产化学与工程国家民委重点实验室, 广西林产化学与工程重点实验室, 广西林产化学与工程协同创新中心, 广西高校食品安全与药物分析化学重点实验室, 广西 南宁 530008; School of Chemistry and Chemical and Engineering, Guangxi University for Nationalities, Key Laboratory of Chemistry and Engineering of Forest Products, State Ethnic Affairs Commission, Guangxi Key Laboratory of Chemistry and Engineering of Forest Products, Guangxi Collaborative Innovation Center for Chemistry and Engineering of Forest Products, Key Laboratory of Guangxi Colleges and Universities for Food Safety and Pharmaceutical Analytical Chemistry, Nanning 530008, China

**Keywords:** 高效液相色谱, 固相萃取, 金属有机框架, 纤维素气凝胶, 西地那非, 保健品, high performance liquid chromatography (HPLC), solid-phase extraction (SPE), metal-organic frameworks (MOFs), cellulose aerogel, sildenafil, health products

## Abstract

研究建立了一种基于UiO-66-NH_2_@纤维素复合气凝胶的高灵敏度固相萃取新方法,与高效液相色谱法联用用于保健品中西地那非的检测。先将纤维素进行醛基和酰肼基的功能化,然后将两种功能化的纤维素通过交联并负载UiO-66-NH_2_形成复合气凝胶。将此复合气凝胶作为固相萃取的吸附剂使用时,易于收集且不需要外加磁场或者抽真空的辅助作用,操作简单。研究对制备所得的UiO-66-NH_2_@纤维素复合气凝胶进行了X-射线粉末衍射、扫描电镜、红外光谱和N_2_吸附等表征,结果显示UiO-66-NH_2_成功负载于气凝胶的孔道中,纤维素气凝胶掺杂了UiO-66-NH_2_之后其孔道结构变得规整,并且比表面积增大。研究优化了复合气凝胶中UiO-66-NH_2_的负载量对萃取的影响,高比例的负载量有利于西地那非的富集,并且最高的负载率为50%。研究优化了影响西地那非富集效率的实验条件,包括溶液pH、萃取时间、洗脱剂类型、洗脱时间、洗脱体积和离子强度。采用安捷伦Zorbax Eclipse Plus C18色谱柱(150 mm×4.6 mm, 5 μm)进行分离,以含0.1 mol/L三乙胺的磷酸盐水溶液(pH=6.50)-乙腈(30∶70, v/v)为流动相进行洗脱,检测波长为292 nm。在最佳的萃取条件下(pH为9.0,萃取时间为60 min,洗脱剂为乙腈,洗脱液体积为3×2 mL,洗脱时间为40 min),该分析方法的线性范围为10~2000 ng/mL (相关系数*R*^2^=0.9949),检出限(LOD, *S/N*=3)为2.85 ng/mL,富集因子为59.17。将该方法用于保健品中西地那非的萃取,所得回收率为74.93%~89.12%,相对标准偏差为2.8%~5.3%,表明了方法的回收率和精密度良好,说明了该方法具有应用于保健品中西地那非日常检测的潜力。

## Abstract (en)

Sildenafil is a prescription drug used to treat pulmonary hypertension and erectile dysfunction. However, the illegal addition of sildenafil to health supplements may be hazardous to human health. Therefore, it is imperative to develop a method for the detection of sildenafil in health products. Solid-phase extraction (SPE) is typically used for the separation, purification, and enrichment of samples, with the aim of reducing the matrix interference of the samples and improving the detection sensitivity. SPE is widely employed in the detection of trace compounds in complex samples. Metal-organic frameworks (MOFs) are hybrid crystalline materials composed of metal ions and organic ligands. MOFs offer the advantages of a large specific surface area, permanent nanopores, good stability, and pore controllability. Owing to their advantages, researchers have attempted to use MOFs for SPE; however, it is difficult to collect MOFs in the powder form when they are directly used in SPE, necessitating the use of a substrate material to fix the MOFs. Hence, this study proposes a novel SPE method with high sensitivity that employs a UiO-66-NH_2_@cellulose hybrid aerogel, for the detection of sildenafil in health products. First, UiO-66-NH_2_ was synthesized by a hydrothermal method. Cellulose nanocrystal (CNC) was modified with an aldehyde group to obtain CNC-CHO, and carboxymethyl cellulose (CMC) was modified with a hydrazide group to obtain CMC-NHNH_2_. Subsequently, UiO-66-NH_2_ was added to the CNC-CHO solution, mixed with the CMC-NHNH_2_ solution, and then crosslinked to load UiO-66-NH_2_ and form a hybrid aerogel as a bulk adsorbent. This bulk hybrid aerogel could be collected easily without extra force for use in SPE. The synthesized hybrid aerogel was characterized by X-ray powder diffraction, scanning electron microscopy, Fourier transform-infrared (FT-IR) spectroscopy, and nitrogen gas adsorption/desorption. The results showed that UiO-66-NH_2_ was successfully loaded on the pore surface of the hybrid aerogel, which made the pore of aerogel become more regular and a larger surface area. Investigation of the loading amount of UiO-66-NH_2_ in the hybrid aerogel revealed that a higher amount of UiO-66-NH_2_ could yield better extraction efficiency. The highest amount of UiO-66-NH_2_ that could be loaded in the hybrid aerogel was 50%. The experimental conditions affecting the enrichment of sildenafil were optimized, and determined to be the following: pH of the sample, 9.0; extraction time, 60 min; eluent, acetonitrile; elution time, 40 min; elution volume, 3×2 mL; salt ion concentration, 0. Separation was performed on an Agilent Zorbax Eclipse Plus C18 column (150 mm×4.6 mm, 5 μm) with a phosphate solution containing 0.1 mol/L triethylamine (pH=6.50)-acetonitrile (30∶70, v/v) as the mobile phase. The detection wavelength was set at 292 nm. Under the optimal conditions, the UiO-66-NH_2_@cellulose hybrid aerogel was used as an adsorbent to extract sildenafil in different concentrations. The peak area was proportional to the sildenafil concentration in the range of 10-2000 ng/mL, with limit of detection (LOD, *S/N*=3) of 2.85 ng/mL and enrichment factor of 59.17. The correlation coefficient (*R*^2^) was 0.9950. Compared to previous preconcentration methods for sildenafil, this method offered a wider linear range. Five batches of hybrid aerogels were simultaneously prepared under the same conditions and used to extract sildenafil; the relative standard deviation (RSD, *n*=3) was 1.71%, indicating that the prepared hybrid aerogels offered good reproducibility. The used composite aerogels were freeze-dried again and reused to extract sildenafil; the recovery was still maintained at 85.23% after five extraction cycles, indicating that the UiO-66-NH_2_@cellulose hybrid aerogel had good regeneration ability. The feasibility of the developed method was verified by analyzing five health products. The results demonstrated the presence of 3.01 μg/g sildenafil in one of the products and no sildenafil in the others. The recoveries of this SPE method ranged from 74.93% to 89.12%, with RSDs in the range of 2.8%-5.3%, proving the feasibility of this analytical method.

西地那非是治疗肺动脉高压和勃起功能障碍处方药中的一种活性化合物^[1,2]^。近年来,西地那非被非法添加到保健品中以达到滋阴补阳的效果^[3]^。但心血管疾病患者和糖尿病患者服用这些药物后会产生严重的副作用^[4]^,因此建立保健品中西地那非的检测方法具有重要意义。目前已采用多种分析方法测定西地那非的含量,包括高效液相色谱法(HPLC)^[5]^、气相色谱-质谱联用法(GC-MS)^[6]^、近红外光谱法(NIR)^[7]^、薄层色谱法(TLC)^[8]^、电化学传感法^[9]^等。其中,高效液相色谱法具有速度快、重复性好、操作自动化、分析精度高等优点,是目前测定西地那非的首选方法。但保健品成分复杂,西地那非添加量低,样品不能直接用高效液相色谱法检测而需要进行预处理。

固相萃取(SPE)具有效率高、重现性好、易于与其他仪器结合等优点,是目前样品处理的首选方法。固相萃取的核心是吸附剂的选择,它直接影响萃取效率。目前,用于西地那非固相萃取的吸附剂主要为分子印迹聚合物^[10]^和功能化磁性材料^[11]^。事实上,固相萃取的吸附剂类型仍有发展的空间。金属有机骨架材料(metal-organic frameworks, MOFs)是由金属离子和有机配体组成的杂化结晶材料,其中MOFs材料具有比表面积大、纳米孔隙持久、稳定性好、孔隙可控性好等优点^[12]^,已广泛应用于分离^[13]^、储气^[14]^、传感^[15]^、催化^[16]^等领域,因此是一种具有广阔应用前景的固相萃取吸附剂。但如果将MOFs粉末直接用于固相萃取时,其粉末不易于收集,因此需要基底材料来固定MOFs。常用的基底材料是磁性材料,但其制备步骤往往比较复杂。

气凝胶是由有机、无机或混合分子通过溶胶-凝胶工艺和适当的干燥技术制成的材料总称。气凝胶有很多种,包括无机气凝胶、有机气凝胶、碳质气凝胶和纤维素气凝胶。其中,纤维素气凝胶具有比表面积大、可回收性和生物降解性好等优点^[17]^,具有广阔的应用前景。Karadagli等^[18]^制备了块状纤维素气凝胶作为绝缘材料。Cai等^[19]^制备了纳米纤维素微球,并在纳米纤维素微球中培养NIH 3T3细胞。Zhu等^[20]^制备了MOFs与纤维素纳米晶的复合气凝胶,成功用于重金属离子的去除。然而至今尚未有将MOF@纤维素复合气凝胶用于固相萃取的研究报道。

本文报道了溶胶-凝胶法制备UiO-66-NH_2_@纤维素复合气凝胶,并建立了以UiO-66-NH_2_@纤维素复合气凝胶为固相萃取吸附剂的固相萃取结合高效液相色谱检测西地那非的方法。首先,将纤维素纳米晶(CNC)经过醛基修饰得到CNC-CHO以及将羧甲基纤维素(CMC)经过酰肼基修饰得到CMC-NHNH_2_;所得的CNC-CHO和CMC-NHNH_2_交联反应负载UiO-66-NH_2_材料制备得到UiO-66-NH_2_@纤维素复合气凝胶。该UiO-66-NH_2_@纤维素复合气凝胶应用于固相萃取,解决了直接使用MOFs粉末时难于收集的问题。同时,UiO-66-NH_2_@纤维素复合气凝胶的分离不需要借助辅助工具。该方法线性范围宽,重复性和回收率好,已成功应用于实际样品。

## 1 实验部分

### 1.1 仪器、试剂与材料

X射线粉末衍射仪(XRD, D8 advance, Bruker,德国);红外光谱仪(FT-IR, Nicolet Is 10, Thermo Fisher,美国);扫描电子显微镜(SEM, SUPPRA 55 Sapphire, Carl Zeiss AG,德国);比表面分析仪(BET, ASAP2460, Micromeritics,美国)。

西地那非(纯度99%)购于上海提坦科技有限公司。CNC购于上海闪思科技有限公司。CMC(*M*_r_250000)、甲醇、乙醇、丙酮、醋酸、高碘酸钠(NaIO_4_)、己二肼(ADH)、羟基琥珀酰亚胺(NHS)、1-(3-二甲氨基丙基)-3-乙基碳二亚胺盐酸盐(EDC)、2-氨基对苯二甲酸、乙腈(纯度>99%)、*N*,*N*-二甲基甲酰胺(DMF)、二甲亚砜(DMSO)、乙二醇购于上海阿拉丁生化科技有限公司。四氯化锆(ZrCl_4_,纯度99%)由上海麦克林生化有限公司提供。透析袋(截留分子量12000~14000 Da)购于梦怡美生物科技有限公司。去离子水是从Milli-Q纯水系统中获得,并用于制备所有储备和工作溶液。

### 1.2 实验方法

#### 1.2.1 UiO-66-NH_2_的合成

UiO-66-NH_2_的制备是在前人报道^[21]^的基础上进行的:将ZrCl_4_ (0.16 g)和2-氨基对苯二甲酸(0.124 g)溶解于DMF (40 mL)中,密封后在120 ℃下反应24 h,所得固体离心(8000 r/min, 10 min)处理,用DMF和乙醇各洗涤3次。最后产物在80 ℃下真空干燥12 h得到粉末材料。

#### 1.2.2 UiO-66-NH_2_@纤维素复合气凝胶的制备

用CMC-NHNH_2_和CNC-CHO化学交联纤维素气凝胶^[20]^。

制备CMC-NHNH_2_的步骤如下:1.0 g CMC和3.0 g ADH溶解在50 mL水中,依次加入2.5 mL 17.5 mg/mL NHS(溶解于DMSO-H_2_O(1∶1, v/v))和0.6 mL 0.3 g/mL EDC (溶解于DMSO-H_2_O(1∶1, v/v))。用HCl和NaOH调节溶液pH至6.8。将溶液透析,每隔4 h换水,所用水体积为透析液体积的100倍。减压蒸发后,所得黏稠液用水配制成质量分数为1%的CMC-NHNH_2_溶液,并于4 ℃保存。

制备CNC-CHO的方法为:先将1.0 g CNC溶解在50 mL水中,然后加入0.6 g NaIO_4_,搅拌2 h后,迅速加入0.3 mL乙二醇使反应停止。将溶液透析,每隔4 h换水,所用水体积为透析液体积的100倍。最后将CNC-CHO溶液减压蒸发后用水调至质量分数为1%的溶液,并于4 ℃保存。

将100 mg UiO-66-NH_2_加入5 g CNC-CHO溶液(质量分数1%)中。超声5 min后,加入5 g上述CMC-NHNH_2_溶液(质量分数1%),并摇晃混匀。将上述黏稠混合溶液2 g加入离心管中,冷藏6 h,然后冷冻干燥24 h,得到成块海绵状UiO-66-NH_2_@纤维素复合气凝胶。

#### 1.2.3 标准溶液的配制

用乙腈制备1000 μg/mL西地那非标准储备溶液,于4 ℃保存。移取一定量的1000 μg/mL标准储备溶液于容量瓶中,用去离子水逐级稀释后得到1、5、10、25、50、100、200、500、1000、2000、5000 ng/mL西地那非标准溶液。

#### 1.2.4 固相萃取过程

固相萃取过程如图1所示,将UiO-66-NH_2_@纤维素复合气凝胶加入含有25 mL 200 ng/mL西地那非溶液(pH=9.0)的锥形瓶中进行固相萃取。萃取时以120 r/min的转速振荡60 min。提取后,用3 mL乙腈洗脱气凝胶2次,每次洗脱时间为40 min。收集洗脱液,用氮气吹干,再用100 μL乙腈重新溶解。样品经0.22 μm过滤器过滤后,在HPLC-DAD系统进样检测。

**图1:**
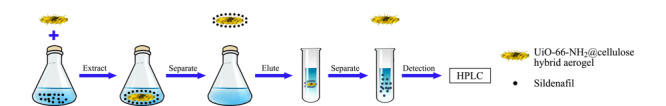
固相萃取过程

#### 1.2.5 样品处理方法

保健品是从网上平台购买的胶囊。胶囊中的粉末样品按以下步骤进行处理^[22]^:充分研磨粉末,并储存在一个深色瓶子里。将1 g样品加入50 mL离心管中,然后加入甲醇50 mL,超声30 min,离心得到上清液,经0.22 μm滤膜过滤。最后收集溶液,用甲醇定容至100 mL。

#### 1.2.6 色谱条件

分析柱为安捷伦Zorbax Eclipse Plus C18色谱柱(150 mm×4.6 mm, 5 μm);柱温为30 ℃;流动相A为含0.1 mol/L三乙胺的磷酸盐水溶液(pH=6.50)-乙腈(30∶70, v/v);等度洗脱;流速为1.0 mL/min;进样体积为20 μL;检测波长为292 nm。

## 2 结果与讨论

### 2.1 材料的表征

通过XRD表征UiO-66-NH_2_、气凝胶和UiO-66-NH_2_@纤维素复合气凝胶3种材料的晶体结构(见图2)。由结果可知,制备的UiO-66-NH_2_的衍射峰与文献^[21]^给予的模拟数据峰位置基本一致;气凝胶为非晶态聚合物,其衍射峰的位置约为25°^[23]^; UiO-66-NH_2_@纤维素复合气凝胶的XRD谱图中,同时存在纤维素气凝胶(弱峰)和UiO-66-NH_2_的衍射峰,表明UiO-66-NH_2_成功负载于纤维素气凝胶内。

**图2:**
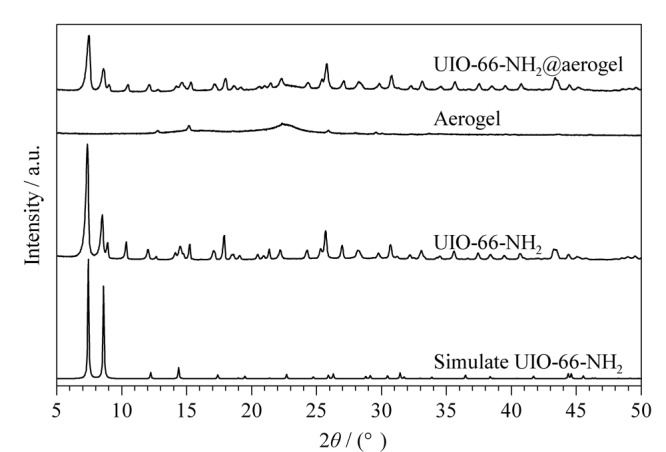
材料的XRD谱图

通过SEM表征材料的形貌。从图3a可看出,UiO-66-NH_2_为八面体,这与之前文献报道的结果一致^[21]^。从图3b可以清楚地看到,未负载UiO-66-NH_2_的纤维素气凝胶的孔壁光滑,孔隙较小。从图3c可以看出,UiO-66-NH_2_@纤维素复合气凝胶的孔壁粗糙,孔壁上附着有UiO-66-NH_2_颗粒,其孔隙排布规整,有整齐的孔道结构。这一现象表明,在纤维素气凝胶中负载UiO-66-NH_2_有利于形成均匀的多孔结构。

**图3:**
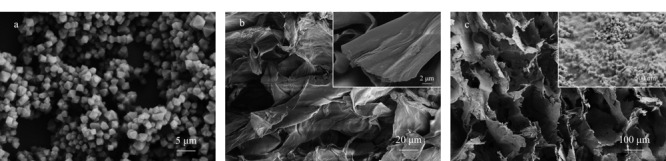
(a) UiO-66-NH_2_、(b)纤维素气凝胶和(c)UiO-66-NH_2_@纤维素复合气凝胶的扫描电镜图

通过氮气吸附和脱附实验研究材料的比表面积和孔径尺寸。由图4a的结果可知,UiO-66-NH_2_的BET比表面积较大(667.86 m^2^/g),纤维素气凝胶的BET比表面积为148.12 m^2^/g,当负载了UiO-66-NH_2_形成复合气凝胶之后,其BET表面积增加到396.67 m^2^/g,较大的比表面积更有利于目标物在材料表面的吸附,从而提高吸附效果。如图4b孔径分布曲线显示,UiO-66-NH_2_、纤维素气凝胶和纤维素复合气凝胶的最可几孔径分别为0.68、3.18和3.18 nm。

**图4:**
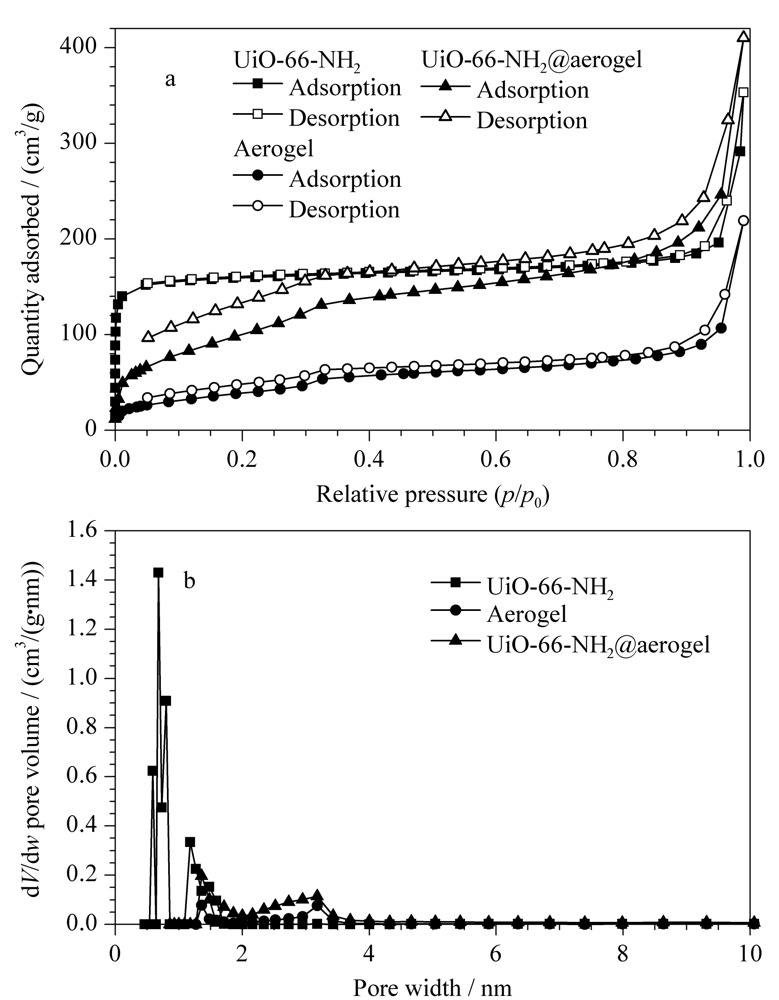
(a)N_2_吸附-解吸等温线和(b)孔径分布图

通过红外光谱表征材料合成过程中的官能团性质(见图5)。改性后的CMC-NHNH_2_在1675 cm^-1^有出峰,说明CMC与ADH反应生成酰胺基团。改性后的CNC-CHO在1723 cm^-1^处出现了一个峰,这是醛基C=O伸缩振动的特征吸收峰,说明CNC的醛基改性是成功的。与CNC-CHO和CMC-NHNH_2_相比,纤维素气凝胶和纤维素复合气凝胶在1065、1637、2976 cm^-1^处具有相似的特征吸收峰,其中CNC-CHO在1723 cm^-1^处的吸收峰消失,表明CNC-CHO与CMC-NHNH_2_成功交联后,醛基被消耗。而在UiO-66-NH_2_@纤维素复合气凝胶的红外谱图中,在1500 cm^-1^处的峰值出现了对应于UiO-66-NH_2_的苯环骨架振动,说明纤维素气凝胶成功负载了UiO-66-NH_2_材料。

**图5:**
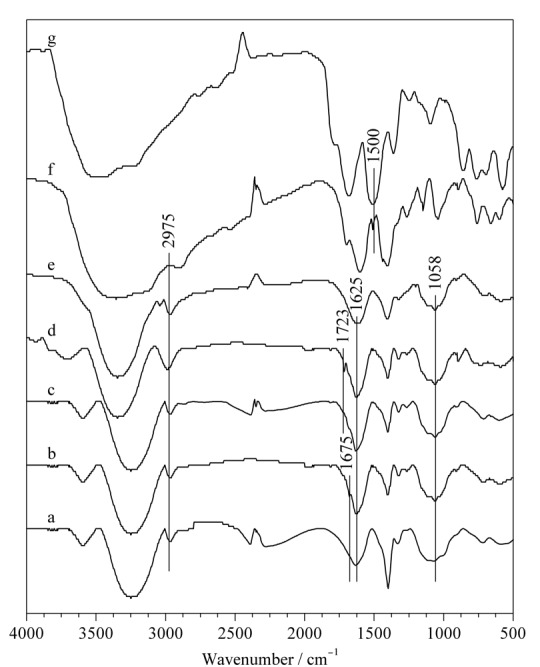
材料的红外光谱图

### 2.2 复合气凝胶中UiO-66-NH_2_掺杂量的优化

在复合气凝胶的制备过程中,以UiO-66-NH_2_材料的质量占纤维素复合气凝胶的总质量比例表示其负载率,分别制备了UiO-66-NH_2_材料的负载率为20%、30%、40%和50%的UiO-66-NH_2_@纤维素复合气凝胶。如果UiO-66-NH_2_材料的负载率大于50%时,在制备过程中混合分散液出现固体沉降。将不同UiO-66-NH_2_材料负载率的复合气凝胶用于对西地那非的固相萃取,随着UiO-66-NH_2_材料负载率的提高,其富集效果越好。因此在后续的萃取应用中,选取50%负载率的复合气凝胶。

### 2.3 固相萃取条件的优化

为了更好地富集分析物,对溶液的pH、萃取时间、洗脱剂种类、洗脱时间、洗脱体积、离子强度等必要参数进行优化。

溶液的pH值会影响目标物的带电性质,从而影响吸附效果。在萃取时间为60 min、洗脱液为乙腈、洗脱液体积为3 mL、洗脱时间为30 min的条件下,在4.0~10.0范围内考察溶液pH对萃取效果的影响。如图6a所示,在pH 4.0~9.0范围内,西地那非的峰面积随着pH的增加而增大,在pH 9.0时达到最大值。这是因为-NH_2_带正电荷,当溶液pH大于西地那非的p*K*_a1_(6.05)^[24]^时,西地那非分子会失去氢离子,倾向于带负电荷。此时,UiO-66-NH_2_@纤维素复合气凝胶与西地那非分子之间的静电相互作用增强。因此选择最佳溶液pH值为9.0。

**图6:**
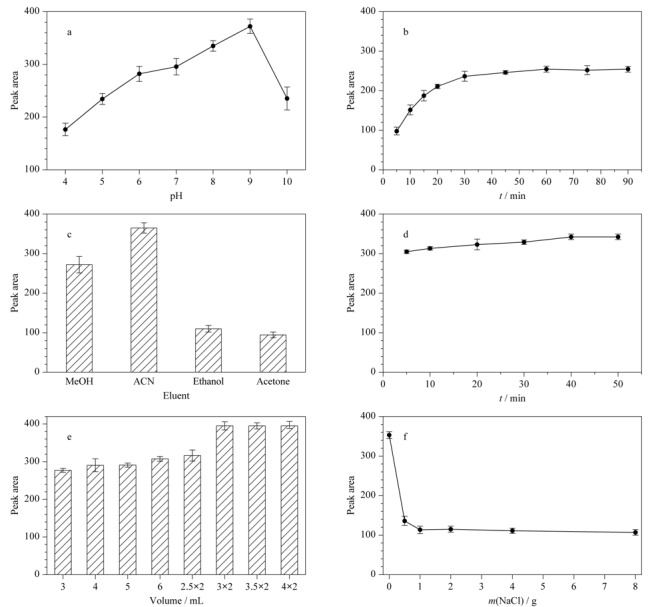
(a)溶液pH、(b)萃取时间、(c)洗脱液种类、(d)洗脱时间、(e)洗脱液体积和(f)离子强度对西地那非(200 ng/mL)峰面积的影响(*n*=3)

萃取是一个吸附过程,达到吸附平衡需要一定的时间,因此对萃取时间进行了优化。在前一步实验条件的基础上(pH为9.0,洗脱液为乙腈、洗脱液体积为3 mL、洗脱时间为30 min),考察萃取时间对萃取效果的影响。从图6b可以看出,西地那非的峰面积随着萃取时间的增加而增大,在60 min时达到平衡,因此在60 min时进行下一步的萃取。

洗脱液的种类、洗脱时间和洗脱液的体积是保证目标分子从复合气凝胶中充分解吸的关键参数,因此必须对这些参数进行优化,以获得更高的富集效率,减少洗脱液的消耗和节省洗脱时间。在pH为9.0、萃取时间为60 min、洗脱液体积为3 mL、洗脱时间为30 min条件下,考察不同溶剂对西地那非解吸效果的影响。从图6c可以看出,在各种洗脱剂中,乙腈的洗脱效果最好,所以我们选择乙腈作为洗脱剂。在pH为9.0、萃取时间为60 min、洗脱液为乙腈、洗脱液体积为3 mL的条件下,考察洗脱时间对萃取效果的影响。图6d显示西地那非的峰面积随着洗脱时间的增加而增大,在40 min时达到平衡,所以洗脱时间选为40 min。在pH为9.0、萃取时间为60 min、洗脱液为乙腈、洗脱时间为40 min的条件下,考察洗脱液体积对萃取效果的影响。实验考察了3、4、5、6 mL乙腈洗脱1次以及用2份2.5、3.0、3.5、4.0 mL乙腈依次洗脱2次(每次时间为40 min)的萃取效果。如图6e表明,当用3 mL洗脱液洗涤2次(即3×2 mL)时,可以获得最好的效果,进一步增加洗脱液体积,峰面积变化不明显,因此选择洗脱液体积为3×2 mL。

为了考察离子强度对富集效果的影响,在pH为9.0、萃取时间为60 min、洗脱液为乙腈、洗脱液体积为3×2 mL、洗脱时间为40 min的条件基础上,实验考察了每100 mL样品溶液中NaCl添加量分别为0、0.5、1.0、2.0、4.0、8.0 g时的萃取效果。从图6f的结果可以看出,添加盐离子后,富集效果降低。可能是因为加盐会使无机盐的水合离子对西地那非分子产生吸附作用,从而影响了UiO-66-NH_2_@纤维素复合气凝胶对西地那非分子的吸附效果。所以最终的实验中选择不添加NaCl。

### 2.4 分析性能研究

根据上述实验结果,采用UiO-66-NH_2_@纤维素复合气凝胶作为吸附剂,在最优条件下对不同浓度的西地那非进行固相萃取。在10~2000 ng/mL范围内,萃取后的西地那非的色谱峰面积(*A*)与西地那非的质量浓度(*C*, ng/mL)成正比,其线性方程为*A*=1.35*C*+87.42,相关系数(*R*^2^)为0.9949,以信噪比(*S/N*)=3确定方法的检出限为2.85 ng/mL,富集因子为59.17(富集因子=(萃取后的线性方程的斜率×1000)/直接检测的线性方程的斜率)。将该方法与已有报道的西地那非的富集方法进行比较(见表1),该方法具有更宽的线性范围,说明该方法具有一定的优越性。

**表1:** 本研究方法与其他已报道方法的比较

Pre-concentration method	Material	Detector	Linear range/ (ng/mL)		LOD/ (ng/mL)	Applications	Ref.
Magnetic solid-phase extraction	Fe_3_O_4_@molecular imprinted polymer	DAD	5	-250	9.49	herbal medicine	[10]
Magnetic solid-phase extraction	Citric acid@Fe_3_O_4_	QTOF	0.5	-25	0.14	urine and plasma	[11]
Magnetic solid-phase extraction	Fe_3_O_4_@nano diamond@graphene oxide	DAD	5	-250	2.2	herbal products	[25]
Liquid-liquid extraction	Ethyl acetate/hexane	UV	10	-1500	5.0	rat plasma	[26]
Solid-phase extraction	Phenyl sorbent	UV	0.2	-20.0	0.066	urine	[27]
Solid- phase extraction	UiO-66-NH_2_@cellulose aerogel	DAD	10	-2000	2.85	health products	this work

### 2.5 吸附剂的再现性和再生使用性能

在相同条件下,平行制备5个批次的复合气凝胶,用于西地那非的固相萃取,所得结果的相对标准偏差(RSD, *n*=3)为1.71%,表明所制备的复合气凝胶具有良好的批次重复性。将同一个使用过的复合气凝胶经过溶剂洗脱后再次冻干,再将其用于西地那非的固相萃取,经过5次的重复萃取后,所得回收率为第一次结果的85.23%,说明制备的UiO-66-NH_2_@纤维素复合气凝胶具有良好的再生使用性能。

### 2.6 实际样品检测

在网上购买了5种保健品作为实际样品,通过对实际样品的分析,验证方法的可行性,所得结果如图7和表2所示。从结果可知,在4号样品的保健品中检测出西地那非的含量为3.01 μg/g(平均值),其他保健品中均未有西地那非检出。对实际样品进行加标回收检测,加标水平分别为5 μg/g和50 μg/g,所得的加标回收率为74.93%~89.12%, RSD为2.8%~ 5.3%,这些结果证明了该分析方法具有良好的重复性和回收率,具有作为日常应用中西地那非检测的潜力。

**图7:**
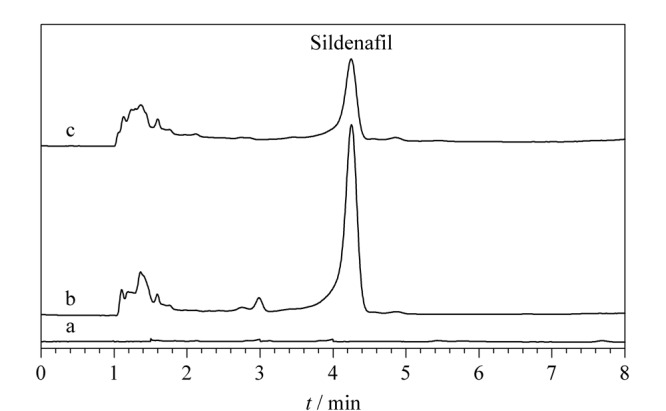
西地那非(200 ng/mL)标准溶液在萃取(a)前、(b)后,以及(c)阳性保健品的色谱图

**表2:** 保健品中西地那非的测定结果

Sample	Added/ (μg/g)	Found/ (μg/g)	Recovery/ %	RSD (*n*=3)/%
Health product 1	0	-	-	-
	5	3.99	79.86	5.30
	50	42.46	84.92	1.80
Health product 2	0	-	-	-
	5	3.76	75.13	4.66
	50	42.59	85.18	3.79
Health product 3	0	-	-	-
	5	3.81	76.2	3.29
	50	43.60	87.20	2.84
Health product 4	0	3.01	-	3.18
	5	7.06	80.6	5.16
	50	47.56	89.12	3.00
Health product 5	0	-	-	-
	5	3.75	74.93	4.96
	50	43.67	87.34	2.98

-: not detected.

## 3 结论

本实验建立了一种基于UiO-66-NH_2_@纤维素复合气凝胶作为固相萃取吸附剂的固相萃取方法。合成的UiO-66-NH_2_@纤维素复合气凝胶具有均匀的多孔结构和较高的比表面积。该固相萃取方法与高效液相色谱联用检测西地那非,具有线性范围宽、检出限低、富集因子高的优点。该技术成功应用于保健品中西地那非的测定,回收率较好,证明UiO-66-NH_2_@纤维素复合气凝胶在西地那非食品样品的预处理应用中具有良好的潜力。
